# Asymmetry of nucleotide substitutions in tRNAs indicates common descent of modern organisms from a thermophilic ancestor

**DOI:** 10.18699/vjgb-25-116

**Published:** 2025-12

**Authors:** I.I. Titov

**Affiliations:** Institute of Cytology and Genetics of the Siberian Branch of the Russian Academy of Sciences, Novosibirsk, Russia Kurchatov Genomic Center of ICG SB RAS, Novosibirsk, Russia

**Keywords:** evolution, thermophile, mutations, tRNA, transition matrix, last universal common ancestor, эволюция, термофил, мутации, тРНК, матрица перехода, последний универсальный общий предок

## Abstract

The nature of the last universal common ancestor (LUCA) of all living organisms remains a controversial issue in biology. There is evidence of both thermophilic and mesophilic LUCA origin. The increasing complexity of the cellular apparatus during the evolution from early life forms to modern organisms could have manifested itself in long-term evolutionary changes in the nucleotide composition of genetic sequences. This work is devoted to the identification of such trends in tRNA sequences. The results of an evolutionary analysis of single-nucleotide substitutions in tRNAs of 123 species from three domains – Bacteria, Archaea and Eukaryota – are presented. A universal vector of directed evolutionary change in tRNA sequences has been discovered, in which substitutions of guanine (G) to adenine (A) and cytosine (C) to uracil (U) occur more frequently than the reverse. The most striking asymmetry in the number of substitutions is observed in the following transitions: a) purine-to-purine, where G→A outnumbers A→G, b) pyrimidine-to-pyrimidine, where C→U outnumbers U→C, and c) purine-to-pyrimidine and vice versa, where G→U outnumbers U→G. As a result, tRNAs could lose “strong” three-hydrogen-bond complementary pairs formed by guanine and cytosine and fix “weak” two-hydrogen-bond complementary pairs formed by adenine and uracil. 16 out of 20 tRNA families are susceptible to the detected change in sequence composition, which corresponds to the significance level p = 0.006 according to the one-sided binomial test. The identified pattern indicates a high GC content in the common ancestor of modern tRNAs, supporting the hypothesis that the last universal common ancestor (LUCA) lived in a hotter environment than do most contemporary organisms

## Abstract (ru)

Природа последнего универсального общего предка (last universal common ancestor, LUCA) всех ныне живущих организмов до сих пор остается актуальной проблемой биологии. Существуют свидетельства в пользу того, что LUCA был как термофилом, так и мезофилом. Усложнение клеточного аппарата в ходе эволюции от ранних форм жизни к современным организмам могло проявиться в долговременных эволюционных изменениях нуклеотидного состава генетических последовательностей. Выявлению подобных тенденций в последовательностях тРНК посвящена эта работа. Представлены результаты эволюционного анализа точечных нуклеотидных замен в тРНК 123 видов трех доменов: Bacteria, Archaea и Eukaryota. Обнаружен универсальный вектор направленного эволюционного изменения последовательностей тРНК, при котором замены гуанина (G) и цитозина (С) на аденин (А) и урацил (U) суммарно происходят чаще обратных. Наиболее ярко асимметрия числа замен наблюдается в следующих переходах: а) между пуринами в преобладании числа замен G→A над числом замен A→G; б) между пиримидинами в преобладании C→U над U→C, а также в) при переходе из пурина в пиримидин и наоборот – в преобладании G→U над U→G. В результате эволюционного процесса тРНК могли терять «сильные» комплементарные пары с тремя водородными связями, формируемые гуанином и цитозином, и фиксировать «слабые» комплементарные пары с двумя водородными связями, образуемые аденином и урацилом. Обнаруженному изменению состава последовательностей были подвержены 16 из 20 семейств тРНК, что соответствует уровню статистической значимости p = 0.006 согласно одностороннему биномиальному тесту. Выявленная закономерность свидетельствует о высоком GC-содержании в последовательности общего предка современных тРНК и, следовательно, подтверждает предположение о том, что самая молодая из гипотетических общих предковых клеток, от которой произошли все ныне живущие организмы (последний универсальный общий предок, LUCA), обитала в более горячей среде, нежели ныне живущие организмы.

## Introduction

Despite extensive research, the nature of the last universal common ancestor (LUCA) of all living organisms remains a pressing problem in biology. According to recent studies (Moody et al., 2024), LUCA arose approximately 4.2 billion years ago and possessed the basic elements of the cellular apparatus of modern prokaryotes (genes and molecular genetic systems for transcription and translation, including tRNAs). There is a debate about whether LUCA was a thermophile (Di Giulio, 2000; Weiss et al., 2016; Moody et al., 2024) or a mesophile (Galtier et al., 1999; Cantine, Fournier, 2017).

The increase in cellular complexity during the evolution from early life forms to modern organisms could have manifested itself in long-term evolutionary changes in the nucleotide composition of genetic sequences. Thus, in the work (Jordan et al., 2005), using the method of unrooted parsimony (Rickert et al., 2025), patterns of systematic unidirectional changes in the amino acid composition of proteins during their evolution from ancestral forms were identified: an increase in the content of the amino acids Cys, Met, His, Ser and Phe due to a decrease in the content of the amino acids Pro, Ala, Glu and Gly. In the work (Galtier et al., 1999), a comparison of LUCA ribosomal RNAs and those of modern species based on GC content was conducted, the results of which were subsequently criticized (Di Giulio, 2000). Of interest is the work (Men et al., 2022), in which fragments of LUCA ribosomal RNAs (16S, 5S, and 23S rRNA) that are evolutionarily conserved in modern sequences and correspond to sites of rRNA interaction with ribosome proteins were reconstructed. However, this study examined rRNA nucleotide sequences in the binary purine-pyrimidine code and, therefore, did not assess the G/C content of the RNA. Therefore, evolutionary changes in the RNA nucleotide composition from LUCA to modern species have not been definitively established.

In this regard, it seemed interesting to study long-term trends in changes in the nucleotide composition of RNA sequences, namely tRNA molecules, which are the most important element of translation systems in all organisms.

In our study, we examined the molecular evolution of 20 isoacceptor tRNA families, each of which mediates the transfer of a specific amino acid during translation. These tRNA families were analyzed for 123 organisms from three domains: Bacteria, Archaea and Eukaryota

Phylogenetic analysis was performed using the unrooted parsimony method (Jordan et al., 2005). Single nucleotide substitutions were identified that became fixed in tRNAs during their evolution from ancestral sequences to modern ones, and it was shown that substitutions of guanine (G) or cytosine (C) for adenine (A) or uracil (U) are fixed more often than substitutions of A or U for G or C. This shapes a view of predominantly unidirectional evolutionary change of tRNA sequences, during which they lost “strong” complementary pairs with three hydrogen bonds formed by guanine and cytosine, and fixed “weak” complementary pairs with two hydrogen bonds formed by adenine and uracil. This feature was characteristic of 16 of the 20 tRNA families, with a significance level of p < 0.006 according to the one-sided binomial test.

## Materials and methods

The tRNA nucleotide sequences of three domains (Bacteria, Archaea and Eukaryota) were taken from a curated database presented in the paper (Sprinzl et al., 1998, Supplementary Material S1)1. The database contained an alignment of tRNA sequences “most compatible with the tRNA phylogeny and known three-dimensional structures of tRNA” (Sprinzl et al., 1998). Each tRNA was assigned to its amino acid by the database authors.


Supplementary Materials are available in the online version of the paper: https://vavilovj-icg.ru/download/pict-2025-29/appx41.zip


The procedure for generating a sample of nucleotide sequences for evolutionary analysis was as follows. 1) For each of the 123 organisms, 20 tRNA groups were considered. Each group included a tRNA interacting with one of the 20 amino acids. Possible horizontal transfer (Soucy et al., 2015), as well as transitions between groups as a result of remodeling (a change in the isoacceptor group as a result of an anticodon change, for which only about 20 cases are currently known

(Bermudez-Santana et al., 2010; Velandia-Huerto et al., 2016; Romanova et al., 2020)) were not considered. 2) For each position of the nucleotide sequences of this group corresponding to a specific organism and amino acid, the frequencies of four nucleotides were calculated, and the nucleotide with the highest frequency was assigned to the position in question; considering all positions of the sequences of the group, a consensus sequence of the tRNA group was constructed. 3) For a consensus sequence corresponding to a particular group of tRNAs, its similarity to each of the nucleotide sequences of the multiple alignment included in the group under consideration was assessed, and the sequence closest to the consensus was selected from this group.

**Fig. 1.**
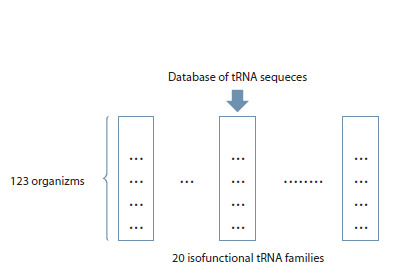
Scheme of building the sample from the tRNA sequence database

Thus, a sample of tRNA nucleotide sequences for evolutionary analysis was formed, containing 20 × 123 = 2,460 typical tRNA sequences (Fig. 1). Each sequence in this sample was most typical for one of the isofunctional tRNA families of a given organism (out of 123).

**Fig. 2.**
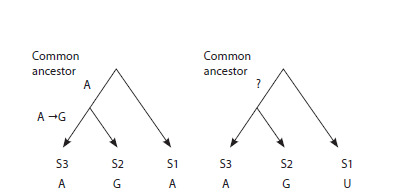
Search for nucleotide substitutions using the unrooted maximum parsimony method on the simplest trees of three closest tRNAs. The identified A→G substitution in the group of two closest relatives, S2 and S3, is shown on the left, and the uninformative substitution is shown on the Fig. 1. Scheme of building the sample from the tRNA sequence database. right.

Following (Jordan et al., 2005), identification of nucleotide substitutions recorded during the evolution of the nucleotide sequences of each isofunctional tRNA family was carried out based on the unrooted maximum parsimony method on phylogenetic trees with three vertices (Fig. 2) using the Dnapars program (Phylip package, Phylip, https://phylip web.github. io/phylip).When analyzing a specific family of isoacceptor tRNAs, the following procedure was performed. For each S1 nucleotide sequence of 123 tRNA sequences in the family, the closest (in terms of similarity) S2 nucleotide sequence was identified, followed by the closest S3 sequence to S2 (Fig. 2), so that S2 and S3 formed a pair of closest relatives. This resulted in the formation of a phylogenetic triad in which S1 was the “outgroup” relative to the pair S2 and S3.

The unrooted maximum parsimony method assumes that if a nucleotide is found at a certain position in the sequence that is identical in S1, S2 and S3, then this nucleotide was present at the same position in the tRNA in the common ancestor of S1, S2 and S3. If, however, a different nucleotide is observed in S3, then a single nucleotide substitution occurred along the branch leading to S3. If all three nucleotides were different, then, following (Jordan et al., 2005), this position was considered uninformative and excluded from consideration. This method does not require stationarity and reversibility of the evolutionary process (Klopfstein et al., 2015).

## Results

Following the approach of (Jordan et al., 2005) and considering nucleotide changes between the sequences of the closest ancestors and descendants, we constructed a mutational transition matrix for each of the 20 aligned tRNA families. Table 1 shows an example of such a matrix for the tRNACys family. Off-diagonal elements Mi, k (i, k = 1,…,4) characterize the total number of single substitutions in the tRNACys sequences of nucleotide i to nucleotide k. Diagonal elements Mi, k correspond to conserved positions. Rows and columns with gaps in the alignments (–) mainly corresponded to the variable loop region and were omitted for quantitative assessments.

**Table 1.**
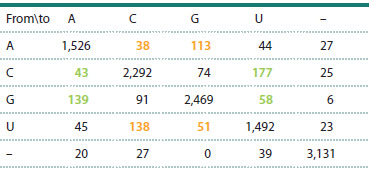
Matrix of the number of single-nucleotide substitutions in tRNACys sequences Note. Here and in Tables 2 and 3: green indicates the number of substitutions of “strong” nucleotides (G and C, which form complementary pairs with three hydrogen bonds) with “weak” nucleotides (A and U, which form complementary pairs with two hydrogen bonds). Yellow indicates the number of substitutions of “weak” nucleotides A and U with “strong” nucleotides G and C. The column marked with a “–” sign indicates the number of substitutions at alignment positions corresponding to deletions.

Table 1 shows that among the nucleotide substitutions identified for the tRNACys family, the most frequently observed were transitions, i. e. substitutions between purines (NG→A = 139 and NА→G = 113) and between pyrimidines (NC→U = 177 and NU→C = 138).

It is noteworthy that the number of substitutions of “strong” nucleotides with “weak” ones (G→A, G→U, C→A, C→U), which is 417, exceeds the number of substitutions of “weak” nucleotides with “strong” ones (A→G, A→C, U→C, U→G), which is 340. This indicates an evolutionary trend toward a decrease in the G/C content of tRNAs in favor of an increase in the A/U content. The effect we identified, described above, was termed nucleotide substitution asymmetry.

We arrive at qualitatively similar conclusions by examining mutational transitions in the tRNAGlu family (Table 2). In this family, the number of substitutions of “strong” nucleotides with “weak” ones is 454, and the number of substitutions of “weak” nucleotides with “strong” ones is 302.

**Table 2.**
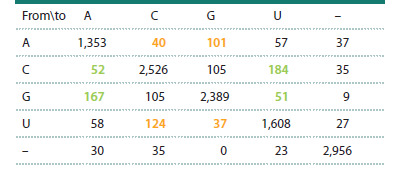
Matrix of the number of single-nucleotide substitutions in tRNAGlu sequences

A similar analysis was performed for all 20 isoacceptor tRNA families (Supplementary Material S2). Next, we estimated the asymmetry effect for all isoacceptor tRNA families. For this purpose, we calculated a general substitution matrix by summing the corresponding elements of all 20 isoacceptor tRNA family matrices (Supplementary Material S2). For all tRNAs, the number of identified single substitutions was 24,653, and the number of uninformative substitutions was 2,083

The diagonal elements of the resulting matrix (Table 3) characterize the average nucleotide composition of tRNAs from the studied species: 32.9 % (G), 27.8 % (C), 21.0 % (U), 18.3 % (A), as well as the content of “strong” G + C nucleotides (60.7 %) and “weak” ones (39.3 %). Transitions are represented by four out of the twelve off-diagonal elements. The proportion of transitions in the total number of substitutions was 56 %.

**Table 3.**
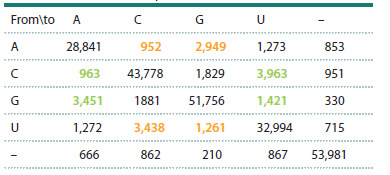
Matrix of the number of nucleotide substitutions identified by the unrooted parsimony method for tRNAs, summarized for all isoacceptor families

As in most partial matrices for individual families of isoacceptor tRNAs (see, for example, Tables 1 and 2), in Table 3, the number of substitutions of “strong” nucleotides with “weak” ones (shown in green) exceeds the number of substitutions of “weak” nucleotides with “strong” ones (marked in yellow): cf. NG→A = 3451 and NА→G = 2949, NC→U = 3963 and NU→C = 3468, NG→U = 1421 and NU→G = 1261, NC→A = 963 and NА→C = 952.

To quantitatively assess the asymmetry of substitutions AF→Z, the relative difference was calculated, defined as the doubled difference of two values divided by their sum – the number of substitutions between nucleotides F and Z, where F, Z∈(A, U, G, C):

**Formula. 1.**
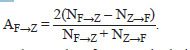
Formula. 1

Table 4 presents the results of AF→Z calculations based on (1) and Table 3. The asymmetry in the number of substitutions was: 0.16 for G→A and A→G; 0.14 for C→U and U→C; 0.12 for G→U and U→G. The remaining transitions were slightly asymmetric: from 0.008 to 0.028 (Table 4).

**Table 4.**
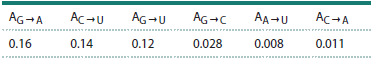
Asymmetry of nucleotide substitutions in tRNAs

Based on Table 3, we can also calculate the balance of losses and gains of ВF for the F-type nucleotide:

**Formula. 2.**
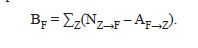
Formula. 2

Table 5 shows the total decrease in the number of “strong” G/C nucleotides in the studied nucleotide sequences of all analyzed tRNA families by 1,198 (714 G + 484 C) due to the evolutionary gain of the same number of weak A/G nucleotides (512 A + 686 U). Considering the total number of G, C, A, and U nucleotides in the studied tRNA sequences, the changes in the number of these nucleotides during the evolution of tRNA families, normalized by their number, were –0.014, –0.011, +0.018, and +0.021 for G, C, A, and U, respectively (Table 5).

**Table 5.**
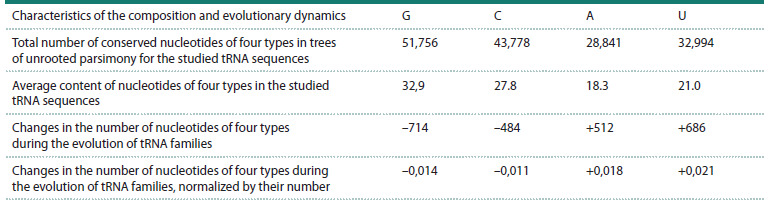
Characteristics of the composition and evolutionary dynamics of the studied nucleotide sequences of all analyzed tRNA families

The nucleotide substitution matrices for all 20 isoacceptor tRNA families are given in Supplementary Material S2. Table 6, obtained from these 20 matrices, shows the arithmetic differences NF→Z – NZ→F (F, Z ∈(А, U, G, C)) between the numbers of all possible types of nucleotide substitutions fixed in the evolution of 20 isoacceptor families of tRNAs. Each variant of the arithmetic difference in the number of F→Z and Z→F substitutions corresponds to a specific column in Table 6. Each row in this table corresponds to a specific isoacceptor family of tRNAs. The last column shows the relative difference in the number of substitutions, AS→W, of “strong” nucleotides, S∈(G, C) with “weak” nucleotides, W∈(A, U), determined by equation (1).

**Table 6.**
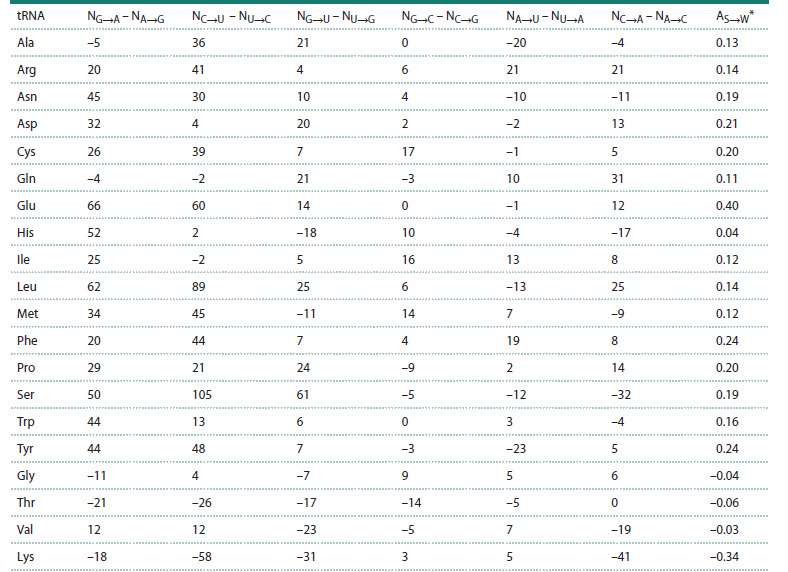
Arithmetic differences NF→Z – NZ→F (F, Z∈(А, U, G, C)) between the numbers of nucleotide substitutions of all possible types fixed in the process of evolution of 20 isoacceptor families of tRNAs * The last column shows the value of the relative difference in the number of substitutions between “strong” and “weak” nucleotides, AS→W = 2(NS→W – NW→S)/ (NS→W + NW→S), where S∈(G, C), W∈(А, U).

Table 6 shows that 16 tRNA families are characterized by a positive value of the relative difference in the number of substitutions, AS→W > 0. At the same time, four families of tRNAs (bottom lines) are characterized by a negative difference, <0. Of these four families of tRNAs, for three tRNAs (tRNAGly, tRNAThr and tRNAVal), the observed negative trend, i. e. the predominance of W→S substitutions over S→W, is insignificant (–0.06 ≤ AS→W ≤ –0.03), and only for tRNALys, the predominance of W→S substitutions over S→W is pronounced (AS→W = –0.34).

A one-sided binomial test was used to assess the significance of the predominance of positive values AS→W characterizing the relative difference between a) the number of substitutions of “strong” nucleotides with “weak” nucleotides (S→W) and b) the number of substitutions of “weak” nucleotides with “strong” nucleotides (W→S) fixed during the evolution of 20 tRNA families (Lehmann, 2012). In our case, the level of significance was calculated as the probability p of random observation of 16 matrices out of 20 with substitutions in favor of a decrease in the number of “strong” G/C nucleotides: see expression (3). At the same time, it was assumed that the number of recorded substitutions of types S→W and W→S was the same on average.

**Formula. 3.**
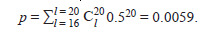
Formula. 3

Using (3), the statistical hypothesis of the asymmetry of evolutionary substitution matrices in the direction of G and C nucleotide loss and A and U nucleotide gain was accepted with a significance level of p <0.006.

## Discussion

Our analysis of the evolution of 20 isoacceptor tRNA families of 123 species of the three domains (Bacteria, Archaea and Eukaryota) from their ancestral forms revealed a tendency to decrease the G/C composition of tRNAs in favor of an increase in the A/U composition. This effect was called the asymmetry of nucleotide substitutions. It consisted in the evolutionary loss of “strong” nucleotides G and C, capable of forming energy-advantageous complementary pairs with three hydrogen bonds, and the gain of “weak” nucleotides A and U, which form less stable complementary pairs with two hydrogen bonds. 16 out of the 20 tRNA families were affected by the detected change in sequence composition, which corresponds to the significance level of p < 0.006 according to the one-sided binomial test.

The results suggest that the last universal common ancestor, LUCA, lived in a hotter environment than currently living organisms; i. e. it was a thermophile or a thermophilic mesophile (moderate thermophile). This conclusion is substantiated by the fact that the content of nucleotides G and C in nucleotide sequences is associated with the optimal temperature of organisms (Dutta, Chaudhuri, 2010), in connection with which genetic macromolecules (DNA, RNA) can be considered as a kind of molecular thermometers, and their G/C content is an indicator of the temperature of the environment.

Early Earth conditions must have determined the energetic, metabolic, biochemical, and environmental features of LUCA. According to (Di Giulio, 2000; Weiss et al., 2016), LUCA lived in hot springs, the high temperature of which facilitates the course of biochemical reactions and molecular genetic processes, but requires thermodynamic and kinetic stability of biomolecular structures, the thermodynamic fluctuations of which are more pronounced the higher the temperature of the environment. Modern thermophiles are adapted to high temperatures due to the high content of nucleotides G and C in the genome (Dutta, Chaudhuri, 2010), which form stronger complementary bonds with each other. And this is especially important for the thermal stability of structural RNAs, including tRNAs.

It should be noted that four out of the 20 families of tRNAs studied in our work do not follow the general trend of losing “strong” nucleotides. The reasons that determined the peculiarities of the evolution of these tRNAs could vary. For example, two families, tRNAGly and tRNAVal, correspond to chemically simple, so-called “Miller” amino acids. Presu- mably, these amino acids were part of the most ancient proteins and the nucleotide composition of their tRNAs could have had time to reach their individual evolutionary equilibrium, albeit different from the average for all tRNAs. However, overall, comparing the G/C composition of tRNAs in organisms living at different temperatures, our results suggest that modern organisms, on average, live in colder environments than LUCA.

## Conclusion

A universal vector of directed evolutionary change in tRNA sequences has been discovered, in which the substitution of guanine (G) and cytosine (C) with adenine (A) and uracil (U) in total occurs more often than the reverse. As a result of the evolutionary process, tRNAs could lose “strong” complementary pairs with three hydrogen bonds, formed by guanine and cytosine, and fix “weak” complementary pairs with two hydrogen bonds, formed by adenine and uracil. 16 out of the 20 tRNA families were affected by the detected change in sequence composition, which corresponds to the level of statistical significance p = 0.006 according to the one-sided binomial test. This pattern suggests high G/C content in the sequence of the common ancestor of modern tRNAs and, therefore, supports the assumption that the youngest of the hypothetical common ancestral cells, from which all currently living organisms descended (the last universal common ancestor, LUCA), lived in a hotter environment than currently living organisms.

## Conflict of interest

The authors declare no conflict of interest.
